# Lichen Symbiosis: Nature's High Yielding Machines for Induced Hydrogen Production

**DOI:** 10.1371/journal.pone.0121325

**Published:** 2015-03-31

**Authors:** Aikaterini Papazi, Elizabeth Kastanaki, Stergios Pirintsos, Kiriakos Kotzabasis

**Affiliations:** 1 Department of Biology, University of Crete, Voutes University Campus, Heraklion, Crete, Greece; 2 Botanical Garden, University of Crete, Gallos Campus, Rethymnon, Greece; CEA-Saclay, FRANCE

## Abstract

Hydrogen is a promising future energy source. Although the ability of green algae to produce hydrogen has long been recognized (since 1939) and several biotechnological applications have been attempted, the greatest obstacle, being the O_2_-sensitivity of the hydrogenase enzyme, has not yet been overcome. In the present contribution, 75 years after the first report on algal hydrogen production, taking advantage of a natural mechanism of oxygen balance, we demonstrate high hydrogen yields by lichens. Lichens have been selected as the ideal organisms in nature for hydrogen production, since they consist of a mycobiont and a photobiont in symbiosis. It has been hypothesized that the mycobiont’s and photobiont’s consumption of oxygen (increase of COX and AOX proteins of mitochondrial respiratory pathways and PTOX protein of chrolorespiration) establishes the required anoxic conditions for the activation of the phycobiont’s hydrogenase in a closed system. Our results clearly supported the above hypothesis, showing that lichens have the ability to activate appropriate bioenergetic pathways depending on the specific incubation conditions. Under light conditions, they successfully use the PSII-dependent and the PSII-independent pathways (decrease of D1 protein and parallel increase of PSaA protein) to transfer electrons to hydrogenase, while under dark conditions, lichens use the PFOR enzyme and the dark fermentative pathway to supply electrons to hydrogenase. These advantages of lichen symbiosis in combination with their ability to survive in extreme environments (while in a dry state) constitute them as unique and valuable hydrogen producing natural factories and pave the way for future biotechnological applications.

## Introduction

Lichens are the symbiotic phenotype of nutritionally specialized fungi (the mycobiont) that acquire, in an ecologically obligate, mutualistic symbiosis, fixed carbon from a population of minute green algal and/or cyanobacterial cells (the photobiont) [1,2]. Most mycobionts belong to the Ascomycota, whereas only a few species of Basidiomycota form lichens. Concerning photobionts, about 85% of lichen-forming fungi associate with green algae, about 10% with cyanobacteria and about 4% simultaneously with both [3]. Lichen-forming fungi are not a monophyletic group, but a polyphyletic, taxonomically heterogenous assembly of nutritional specialists [4]. The majority of lichen-forming fungi form crustose, often quite inconspicuous thalli on or within the substratum, and only near 25% of lichen mycobionts form shrubby, leaf- or band-shaped, erect or pendulous thalli, usually known as macrolichens [5]. This symbiotic phenotype proved to be so successful that lichens dominate close to 10% of the earth's terrestrial ecosystems, which encompass areas, such as tundra, where higher plants are at their physiological limits.

The biochemical mechanisms of this successful symbiotic phenotype may be an important key in solving the world’s energy problem. It has been known for more than 75 years that, under anaerobic conditions, unicellular green algae and cyanobacteria can metabolize H_2_, either by uptaking it in the dark and using it as an electron donor in the CO_2_-fixation process, or by producing H_2_ in the light [6–9].

There are three known pathways for hydrogen production in green algae: two light-induced, Photosystem II (PSII)-dependent and PSII-independent pathways, and a dark-induced fermentative pathway. The PSII-dependent pathway involves the transport of electrons derived from water splitting to ferredoxin and hydrogenase through Photosystem I (PSI) [6–9]. The PSII-independent pathway depends on the metabolic oxidation of stored organic compounds that is coupled to PSI through the plastoquinone (PQ)-pool and results in both H_2_ production and CO_2_ release [10,11]. Dark fermentation is driven by the anaerobic metabolism of pyruvate, which is catalyzed by one of two enzyme systems: pyruvate formate lyase (PFL) or pyruvate ferredoxin oxidoreductase (PFOR) which transfers electrons to ferredoxin and hydrogenase for hydrogen generation [12].

The main problem in the above-mentioned pathways is that hydrogenase is highly sensitive to O_2_, which irreversibly inactivates the enzyme’s activity within a few minutes [13]. Several attempts have been made to establish anoxic conditions, such as the continuous flow of helium, nitrogen or argon [14], sulfur depleted conditions [15], nitrogen depleted conditions [16], potassium depleted conditions [17] or addition of *meta* substituted dichlorophenols to the culture medium [18,19]. Few attempts tested hydrogen productivity using a combinational system of bacteria (as O_2_ consumers) and algae (as H_2_ producers) [20]. This could be attributed to the extremely higher proliferation of bacterial populations compared to algal growth in the presence of any organic substrate (such as glucose) demanded for increased hydrogen production [19]. Using glucose as a treatment, usually led to the disappearance of the algal strain while the surviving bacteria consumed the glucose without producing hydrogen. Under these circumstances, the idea of using bacteria, to consume oxygen in order to establish the demanded anoxic conditions for algal hydrogen generation, was not effective enough.

The above combinational system was the source of inspiration for us, in using a perfectly symbiotic organism that has already existed in nature for millions of years. Lichen-like fossils were discovered in marine phosphorites of the Doushantuo Formation in South China (approx. 600 MaBP) [21] and as epiphytes in the famous Early Devonian Rhynie Chert beds in Scotland (approx. 460 MaBP) [22,23]. The novelty of our idea that lichens could be the ideal organisms for H_2_ production derives from their symbiotic relationships; they consist of O_2_ consumers (fungus) as well as O_2_ and H_2_ producers (green algae) in a common phenotype and in the absence of competitive exclusion. In a closed system the fungus consumes oxygen, establishes anoxia and ensures the appropriate conditions for effective hydrogen production by it’s photobiont. In other words lichen symbiosis could be viewed as a high yield natural machine for hydrogen production.

In the present contribution we tested the above hypothesis by exploring the capacity of the lichen *Pleurosticta acetabulum* to produce hydrogen under various incubation conditions of glucose concentration, medium factors (volume and composition), temperature levels and light intensities. In addition, to further explore the range of validity of our hypothesis, we tested various lichen species for their capability to produce hydrogen, based on the best hydrogen productivity conditions outlined by *Pleurosticta acetabulum*.

## Results

The oxygen-sensitivity of hydrogenase is the main obstacle in photosynthetic hydrogen production by green algae. Lichens are symbiotic organisms consisting of a mycobiont that consumes oxygen and a photobiont that in the case of green algae has the ability to produce hydrogen in a hermitically closed system. The pilot study was completed utilizing the species *Pleurosticta acetabulum*, because a) it is relatively abundant on Cretan mountains, as at least five replicates in each experimental treatment were required, b) being a macrolichen it is fairly easy to work with, c) it is a green algae lichen, d) it has been confirmed with sequenced data that the photobiont of *Pleurosticta acetabulum* is *Trebouxia arboricola* [24] (GeneBank numbers are also reported in the reference), e) it has never been reported among the cyanolichen species or among the tripartite lichen species which contain both green algal and cyanobacterial symbionts in addition to the lichen-forming fungus [25], f) it has been tested that different parts of the thalli of *Pleurosticta acetabulum*, specifically the apothecial margin, the margin and the center of the thallus, exhibit a homogeneous photobiont population of *Trebouxia arboricola* and not distinguishable populations of photobionts [26] and g) the species exhibits only sexual reproduction of the fungal partner, which indicates an external to the thallus origin of *Trebouxia* (free-living populations of *Trebouxia arbicola*) during the new thalli formation.

There have been no reports of hydrogen production by the algal cells belonging to the genus *Trebouxia* [27]. Thus, this pilot study seems to be the first, concerning the ability of *Trebouxia* lichenized algae to produce hydrogen.

It is worth mentioning that despite the well-established free-living origin of *Trebouxia* algae in the case of *Pleurosticta acetabulum*, it is not known whether these free-living algal cells have escaped from lichen thalli/propagules just for a short period of time before re-lichenization (eg. in case that the fungal hyphae of the propagule die and thus set free the algal cells) or have developed in nature independently of lichen fungi. However, this query is related to the debate about whether or not *Trebouxia* occurs in a “free-living” state [28,29], which is out of the scope of this article.

### Can lichens produce hydrogen?

**Fig 1:**
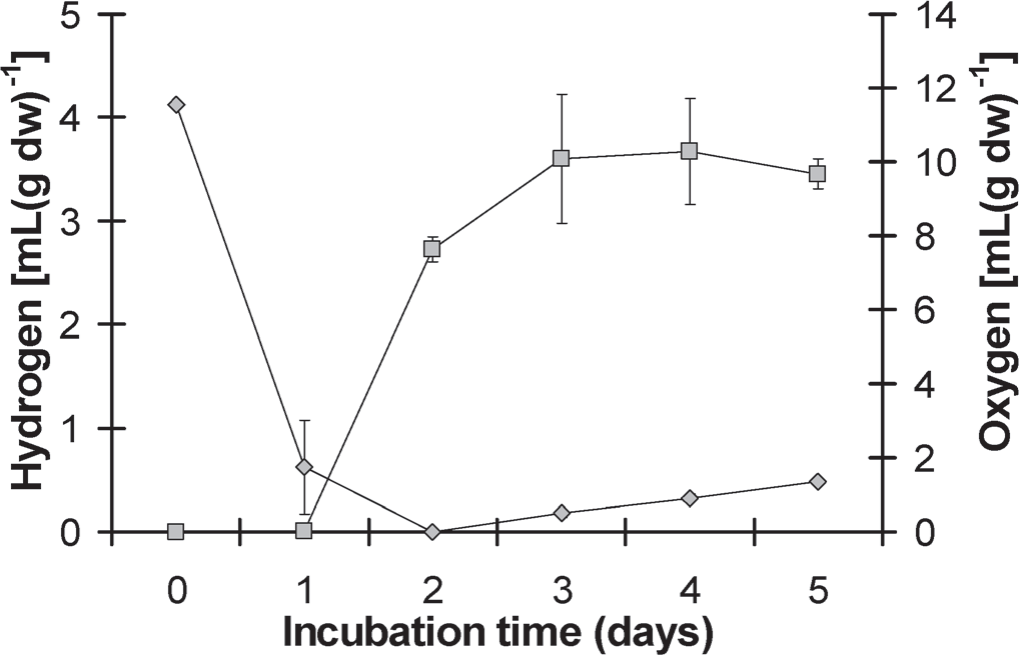
Kinetic of hydrogen production and oxygen consumption of the lichen *Pleurosticta acetabulum*.

Previous experience of our laboratory group in the field of hydrogen production by the green alga *Scenedesmus obliquus* [17–19] led us to use similar initial incubation conditions for testing the hydrogen productivity of the lichen *Pleurosticta acetabulum*. The above decision was based on that only the photobiont part of the lichen (the algae) may have the ability to produce hydrogen and the results were impressively hopeful (Fig. 1). *Pleurosticta acetabulum* functions as oxygen consumer (mainly the mycobiont component) and hydrogen producer (the photobiont component), and as a result can overcome the problem of the inactivation of hydrogenase by the presence of oxygen. It is obvious that in the first 24 hours there was a dramatic drop in the oxygen concentration in the hermitically closed bottles, while at the same time traces of hydrogen were beginning to be detectable (Fig. 1). Under these experimental conditions (analytically described in the section of Materials and Methods) the maximum value of hydrogen production for *Pleurosticta acetabulum* was approximately 3.7 mL H_2_ per g of dry weight of the lichen. This hydrogen production value is extremely high if we consider the extremely low density of green algae that exist in one gram of the lichen’s thallus as compared to the dominant mycobiont.

### Effect of glucose on the hydrogen production of lichens

The exogenously supplied glucose as an organic carbon source has a confirmed positive effect on the hydrogen productivity of green algae [17–19]. Glucose is a commonly used source of carbon and a bidirectional effect on the lichen was expected (an increase in both, the respiration mainly of the mycobiont and the hydrogen production of the phycobiont).

**Fig 2:**
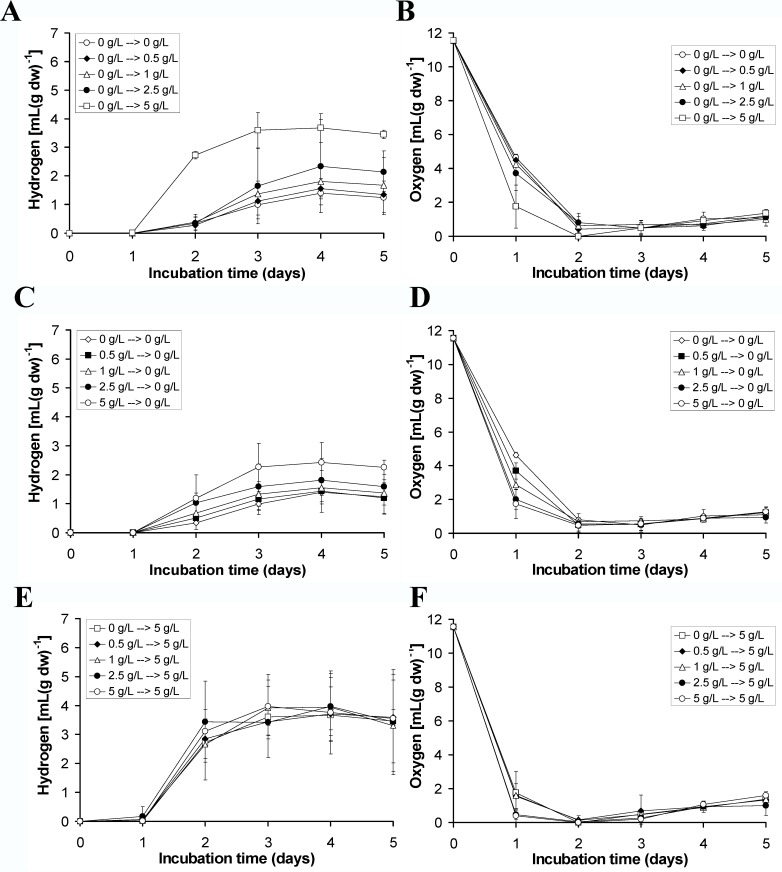
Kinetics of hydrogen production and oxygen consumption in the lichen *Pleurosticta acetabulum*. (A) and (B) regeneration in deionized water (no addition of glucose) and culture medium with 0, 0.5, 1.0, 2.5 or 5.0 g L^-1^ glucose. (C) and (D) regeneration in 0, 0.5, 1.0, 2.5 or 5.0 g L^-1^ glucose and culture medium without glucose. (E) and (F) regeneration in 0, 0.5, 1.0, 2.5 or 5.0 g L^-1^ glucose and culture medium with 5 g L^-1^ glucose.

The influence of glucose on the hydrogen productivity of *Pleurosticta acetabulum* was examined using different concentrations in the regeneration stage, in the culture medium inside the hermitically closed bottles or in both. The tested concentrations of glucose were 0, 0.5, 1.0, 2.5 and 5.0 g L^-1^ and the results are presented in Fig. 2. The hydrogen production appears in the left column, while the corresponding oxygen consumption is on the right.

The treatments in Fig. 2A-B were regenerated in deionized water (no addition of glucose), while the medium used in the hermitically closed bottles contained glucose in the above mentioned gradient concentrations. The rise in glucose concentration led to the expected increase in the oxygen consumption and, as a result to a higher hydrogen production.

Similar results appear in Fig. 2C-D, where the experimental conditions were reversed. In this case, the stage of regeneration took place in the presence of glucose (gradient increase of glucose concentration), while the medium was absent of it. The treatment of using glucose only in regeneration and not in the culture medium resulted in the same trend (higher glucose concentration led to higher oxygen consumption and higher hydrogen production), but the actual hydrogen values were lower compared to the values in Fig. 2A-B. Specifically, the hydrogen productivity in the treatment of 5 g L^-1^ glucose only in the regeneration stage led to 2.5 mL of H_2_ per g dry weight, instead of 3.7 mL of H_2_ per g dry weight that was measured in the case of the 5 g L^-1^ glucose addition only in the medium.

The above results showed that in any case (either in the regeneration stage or in the medium), the presence of glucose was beneficial for the optimization of hydrogen production. Also, the higher the tested glucose concentration, the higher the hydrogen production detected. Those observations were the main reasons for testing the combinational use of glucose in both stages, in the stage of regeneration as well as in the stage of incubation. The results are presented in Fig. 2E-F. The influence of glucose in the regeneration stage was minuscule when glucose existed in the culture medium in the best determined concentration of 5 g L^-1^.

As a result, all the following attempts for the optimization of the lichen’s hydrogen production were completed in deionised water for the regeneration stage and in 5 g L^-1^ glucose for the incubation stage.

### Effect of the culture medium volume on hydrogen production

The above mentioned results evidenced that the addition of glucose, in the culture medium of the hermitically closed bottles, increased the hydrogen generation of the lichens. The volume of the culture medium could also indirectly be a beneficial factor for increasing the hydrogen production. We expected that the larger volume of the liquid culture medium would lead to lower initial available oxygen (in the remaining air space of the 125 mL hermitically closed bottles), allowing for the quicker activation of the hydrogenase enzyme. In addition, we anticipated an increased absorbance of glucose, through the improved contact of the lichen tissue with the medium that would result in higher hydrogen production.

**Fig 3:**
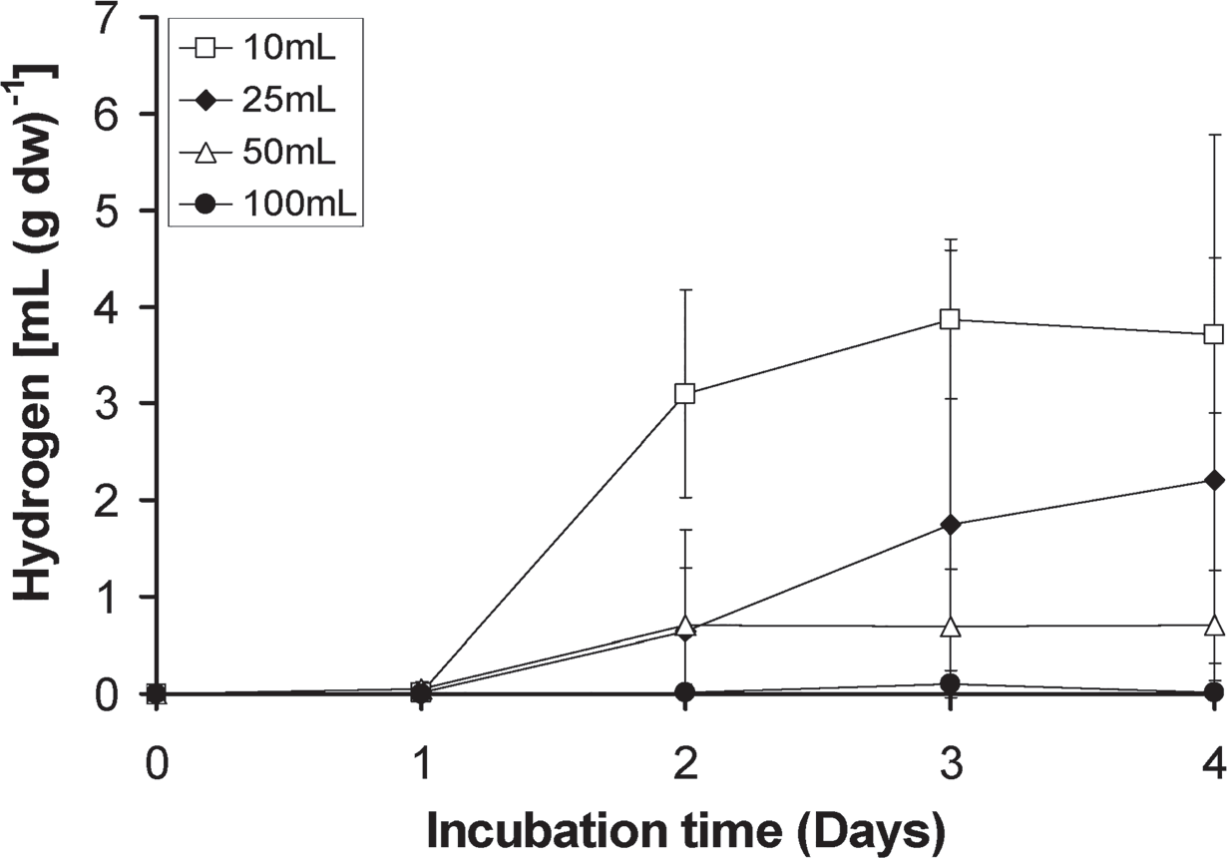
Kinetic of hydrogen production of the lichen *Pleurosticta acetabulum* in several initial volumes of culture medium.

This hypothesis was the main reason for testing the lichen’s hydrogen release in several liquid culture medium volumes (10, 25, 50 and 100 mL). The results for oxygen concentration were the expected ones (increase of medium volume led to decrease of oxygen—data not shown), but the hydrogen productivities based on the hydrogen measurements in the air space were exactly the opposite. The higher the medium volume the lower the hydrogen production measured, as it is shown in Fig. 3. This was mainly due to the partial pressure of the gases in the air-liquid interface, as analytically explained in our previous publication [17]. The increase of the culture medium volume led to faster oxygen depleted conditions, but the partial pressures did not permit the release of the produced hydrogen from the liquid phase to the air phase in order to be detected by the gas chromatographer. During each sampling, where there was a small air decompression (in the upper air phase), air bubbles appeared in the liquid phase (culture medium) which immediately moved to the air phase of the hermitically closed bottle, because of the new pressure balance.

As a result, the volume of 10 mL of culture medium (the one used up to this point) was appropriate for further experimental procedures without changing the conditions of our hermitically closed bottle-systems.

### Effect of the medium composition on hydrogen production

The culture medium plays a crucial role in the lichens’ hydrogen productivity. All the above treatments took place with the well-known medium (for green algae) of Bishop and Senger enriched with 5 g L^-1^ glucose [30]. The used medium could be effective for green algae but not for the lichen as a whole, since they are terrestrial organisms. Therefore, lichens may not be capable of managing the accumulated salts that are present in the culture medium.

**Fig 4:**
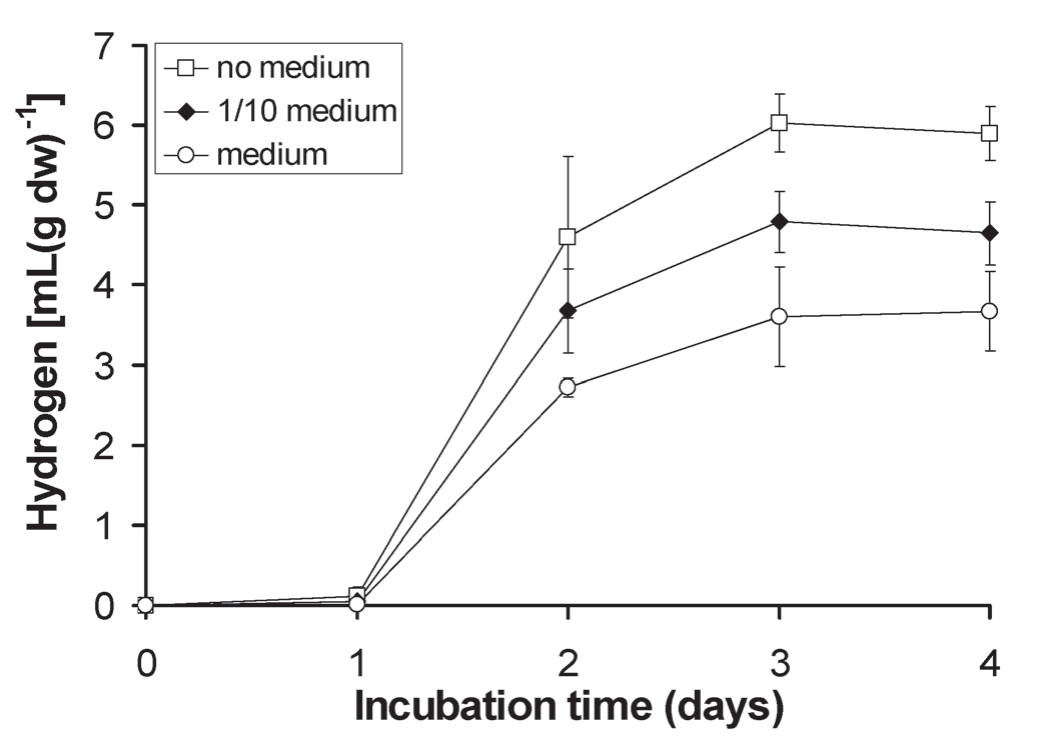
Kinetic of hydrogen production of the lichen *Pleurosticta acetabulum* in several culture mediums.

Different concentrations of the initially used culture medium were examined and the results are presented in Fig. 4. The most interesting observation was that every tested concentration of the growth medium led to lower values of hydrogen production as compared to deionized water with 5 g L^-1^ glucose. Specifically, the lower the concentration of the medium the higher the hydrogen values detected.

As a result, deionized water enriched with 5 g L^-1^ glucose was selected for the following experimental procedures for the optimization of the lichen’s hydrogen generation.

### Effect of temperature on hydrogen production

Temperature is an abiotic parameter that significantly affects the metabolic rates and the activation of enzymes [31,32]. Higher metabolic rates were expected in higher temperatures. However, the rate of an enzyme reaction usually has an optimal temperature. Lower or higher temperatures (than the optimal one) led to reduced efficiencies.

**Fig 5:**
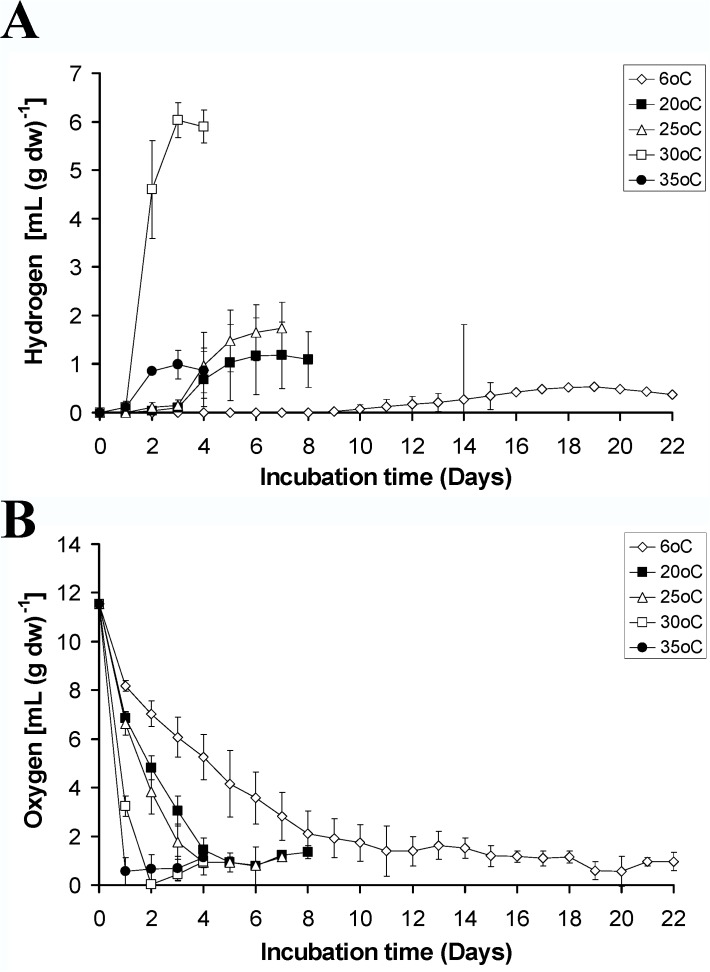
Kinetic of hydrogen production (A) and oxygen consumption (B) of the lichen *Pleurosticta acetabulum* in several temperatures.

The effect of temperature on the hydrogen productivity of *Pleurosticta acetabulum* was examined using five different temperatures (6°C, 20°C, 25°C, 30°C and 35°C). The temperature of 30°C was the one used up to this point. The results for each temperature are presented in Fig. 5A for the hydrogen production and Fig. 5B for the oxygen consumption. As it was expected, the oxygen consumption was higher in increasing gradient temperatures, since there was a corresponding increase in the lichen's metabolism. Higher temperatures led to more intensive respirational rates mainly of the mycobiont and this fact could potentially allow the phycobiont to produce a higher yield of hydrogen. However, the hydrogen production was more beneficial in the temperature of 30°C. Higher temperatures (35°C) led to an earlier detection of hydrogen, but overall lower hydrogen productivities similar to those of 20 and 25°C.

Lower temperatures (6°C) demanded additional incubation time for hydrogen release (after the 9^th^ day) and the detected values were extremely low compared to all the other tested temperatures. The above mentioned observation was mainly due to the lower metabolic rates of the mycobiont component of the lichen that cannot consume the undesirable oxygen (for hydrogenase activation) which consequently leads to the insufficient activation of the hydrogenase enzyme.

As a result, the temperature of 30°C was deemed the best for higher hydrogen productivities and was the one used in the following experimental procedures.

### Effect of light intensity on hydrogen production

The intensity of light is a crucial parameter for photosynthetic organisms [17] and therefore for the phycobiont component of the lichen. Regarding hydrogen production, light intensity affects the oxygen production and as a result the hydrogenase activation.

**Fig 6:**
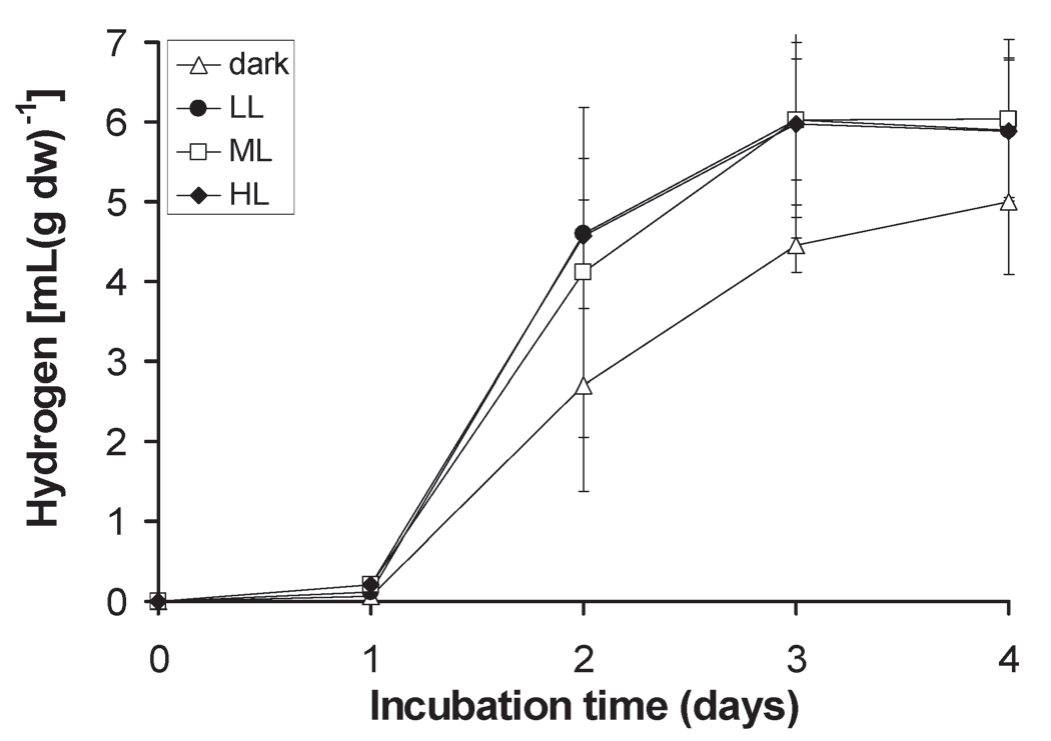
Kinetic of hydrogen production of the lichen *Pleurosticta acetabulum* in several light intensities.

The effect of light intensity on hydrogen generation of the lichen *Pleurosticta acetabulum* was tested using four different light intensities [dark (D): 0 μE, low light (LL): 20–25 μE (the one used up to this point), medium light (ML): 80–100 μE and high light (HL): 200–250 μE]. The kinetics of hydrogen production under the above mentioned light intensities are presented in Fig. 6. Two interesting observations emerged from Fig. 6. Firstly, the hydrogen production of lichens is not exclusively a light induced procedure. Hydrogen is detected both in darkness (possibly through dark fermentation) and in light conditions (PSII-dependent and PSII-independent pathways). Secondly, the hydrogen measured in the light conditions was not affected by the tested intensities (LL, ML and HL), since all the hydrogen values were quite similar.

The actual hydrogen production in the light (approximately 6 mL H_2_ per g dry weight) was higher than the corresponding one measured in the dark (approximately 5 mL H_2_ per g dry weight). The above mentioned difference in hydrogen productivities (measured in dark and light conditions) could be the key for the better understanding of the bioenergetic mechanisms that regulate hydrogen production in lichens.

### Changes in central proteins of the photosynthetic and respirational electron transport chains in light and dark conditions

**Fig 7:**
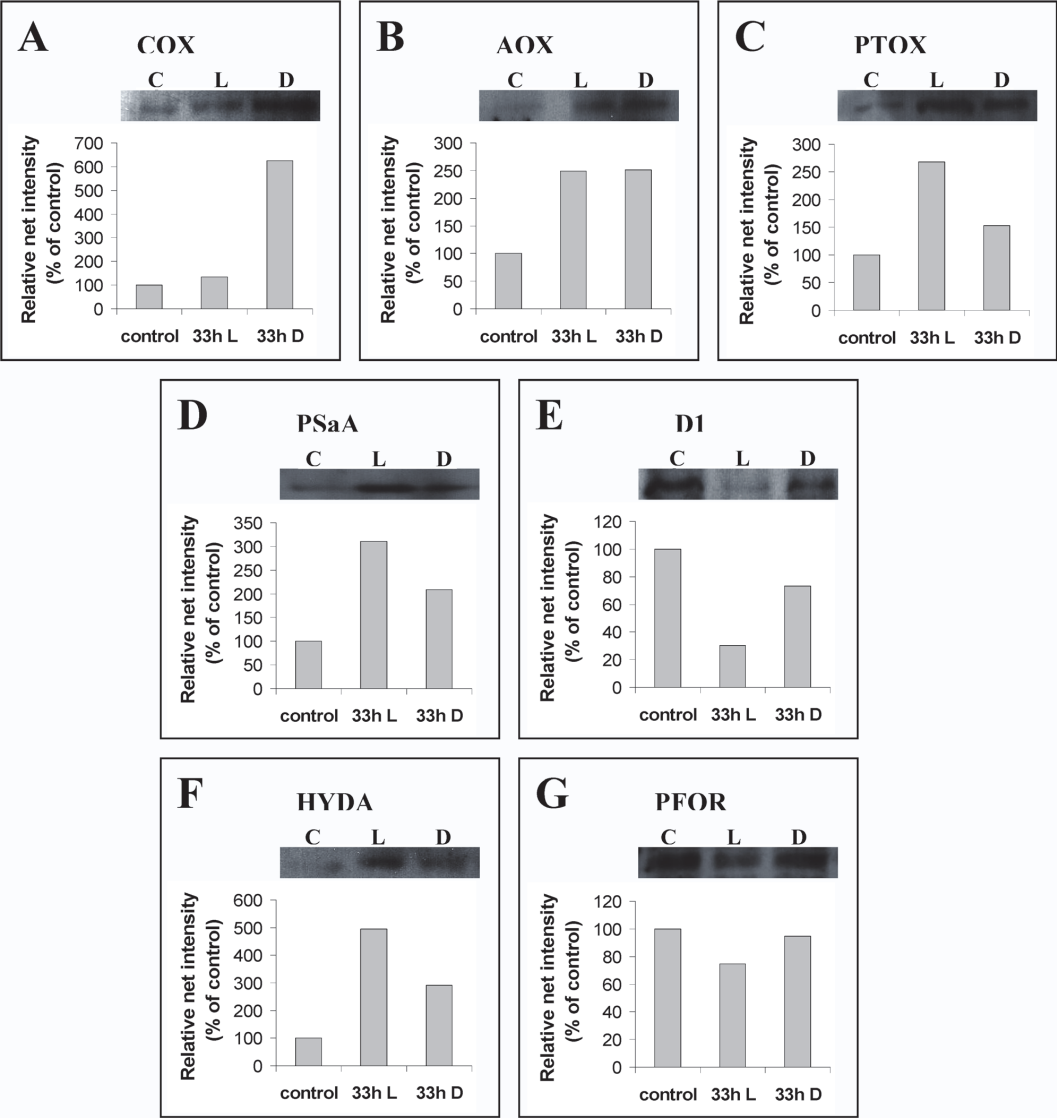
Western blot analysis images and densitometric analysis for (A) cytochromic oxidase (COX), (B) alternative oxidase (AOX), (C) plastid terminal oxidase (PTOX), (D) core protein of PSI (PsaA), (E) core protein of PSII (D1), (F) hydrogenase (HYDA) and (G) pyruvate ferredoxin oxidoreductase (PFOR) after regeneration and after 33 hours of incubation in the hermitically with septum closed bottles. C: Control (after regeneration), L: 33 hours of incubation in light, D: 33 hours of incubation in dark.

Central proteins of the photosynthetic and respirational electron transport chains (Fig. 7) were examined in order to study the hydrogen production pathways of lichens in depth.

There are three known respirational electron transport chains. Two of them are located in mitochondria (cytochromic and alternative pathways) [33,34] and the last one in chloroplasts (chlororespiration) [35–37]. Cytochromic oxidase protein (COX) (Fig. 7A) was chosen for testing the cytochromic pathway, alteranative oxidase protein (AOX) (Fig. 7B) for the alternative pathway and plastid terminal oxidase protein (PTOX) (Fig. 7C) for chlororespiration. Western blot analyses showed an increase in the quantity of all the respirational proteins after 33 hours of incubation compared to the ones analyzed exactly after the regeneration.

As a result, all oxygen scavengers (COX, AOX and PTOX) were fully activated. COX and AOX were more abundant in dark conditions, while at the same time PTOX was more abundant in light, as it was expected. The activation of respirational O_2_ consumers was confirmed by the oxygen decrease (approximately to zero value) in the hermitically closed bottles with the lichen, that were measured using a gas chromatographer (Figs. 1, 2B, D, F and 5B) and was in absolute agreement with the protein abundance (Fig. 7A-C). The establishment of oxygen depleted conditions was the key for the activation of hydrogenase.

The hydrogenase enzyme was fed with electrons through ferredoxin. The origin of electrons could be the splitting of water (PSII-dependent pathway), the reduction of glucose (PSII-independent pathway) and dark fermentation through pyruvate ferredoxin oxidoreductase (PFOR). The examination of PSII took place through the detection of the D1 protein (PSII core protein) (Fig. 7E), the examination of PSI by the PSaA protein (PSI-core protein) (Fig. 7D) and dark fermentation by the abundance of the PFOR protein (Fig. 7G). The western blot analyses showed a decrease in the PSII core protein (Fig. 7E) and in parallel an increase in the PSI core protein (Fig. 7D). These changes were more intensive in light conditions than in dark, as it was expected, and in combination with the over expression of respirational oxidases (COX, AOX and PTOX) created the optimal conditions for increased hydrogen productivity. The above mentioned observations can explain the hydrogen production in light conditions due to the PSII dependent and PSII-independent pathways.

In fact, the hydrogenase protein (Fig. 7F) was more abundant in light compared to dark conditions. However, after dark incubation the hydrogenase protein was more abundant than the protein measured exactly after regeneration (control). The detection and activation of hydrogenase in the dark was a paradox result since the source of electrons to hydrogenase could not come from water splitting (PSII dependent pathway) or the reduction of glucose (PSII independent pathway). This was the main reason for examining the abundance of the PFOR protein that is dominant in the pathway of dark fermentation. It was observed that PFOR (Fig. 7G) was lower in light compared to dark conditions as well as after regeneration. This was mainly due to the fact that in light conditions electrons are directed to hydrogenase from PSII dependent and PSII independent pathways, while in dark conditions, where the above mentioned pathways could not function, the dark fermentative pathway was dominant.

### Is *Pleurosticta acetabulum* the only lichen species that produces hydrogen?

**Fig 8:**
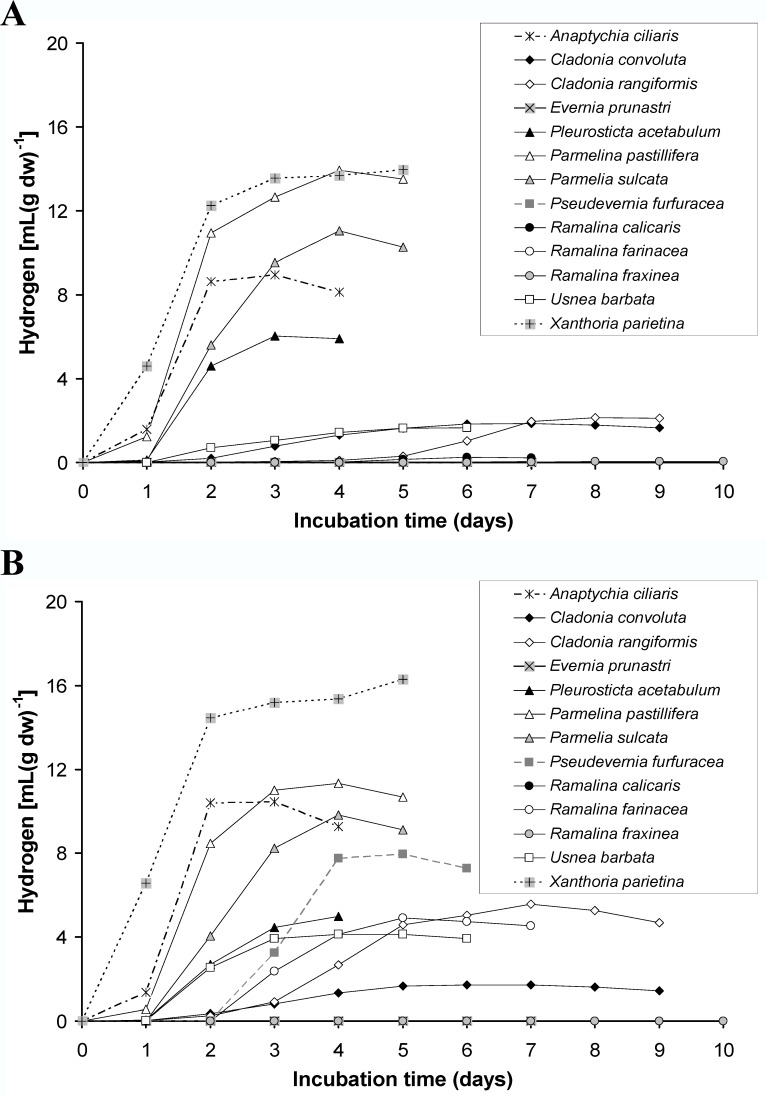
Kinetic of hydrogen production of various lichen species under (A) light and (B) dark incubation.

*Pleurosticta acetabulum* is a lichen species that can produce hydrogen in both light and dark conditions. Is this capability a specific characteristic of the particular lichen species or is it a general aspect of more than one lichen species? The optimal conditions for higher hydrogen productivity of *Pleurosticta acetabulum* were chosen for testing a range of various lichen species for their ability to produce hydrogen in dark and low light conditions (20–25 μE). Briefly, these conditions were regeneration in deionized water and addition of 10 mL sterile deionized water with 5 g L^-1^ glucose in the hermitically with septum closed bottles in a controlled temperature of 30°C. The results are presented in Fig. 8 (8A for light conditions and 8B for dark conditions) and support that lichens in general have the ability to produce hydrogen. Indicative reports concerning the symbiont composition of the tested lichen species are presented in Table 1.

**Table 1:** Green algae as symbiotic photobionts in the studied lichen species.

Lichen species	Green algae	Reference
*Anaptychia ciliaris*	*Trebouxia decolorans*	[44]
*Cladonia convolute*	*Asterochloris*	[45]
*Cladonia rangiformis*	*Asterochloris*	[45]
*Evernia prunastri*	*Trebouxia jamesii*	[46]
*Pleurosticta acetabulum*	*Trebouxia arboricola*	[24,26,47]
*Parmelina pastilifera*	*Trebouxia* sp	[48]
*Parmelia sulcata*	*Trebouxia impressa*	[47]
*Pseudevernia furfuraceae*	*Trebouxia* sp *Trebouxia simplex*	[49,50]
*Ramalina calicaris*	*Trebouxia jamesii*	[46]
*Ramalina farinacea*	*Trebouxia jamesii*	[46]
*Ramalina fraxinea*	*Trebouxia jamesii*	[46]
*Usnea barbata*	*Trebouxia* sp	[51]
*Xanthoria parietina*	*Trebouxia jamesii Trebouxia arboricola*	[44,52]

**Fig 9:**
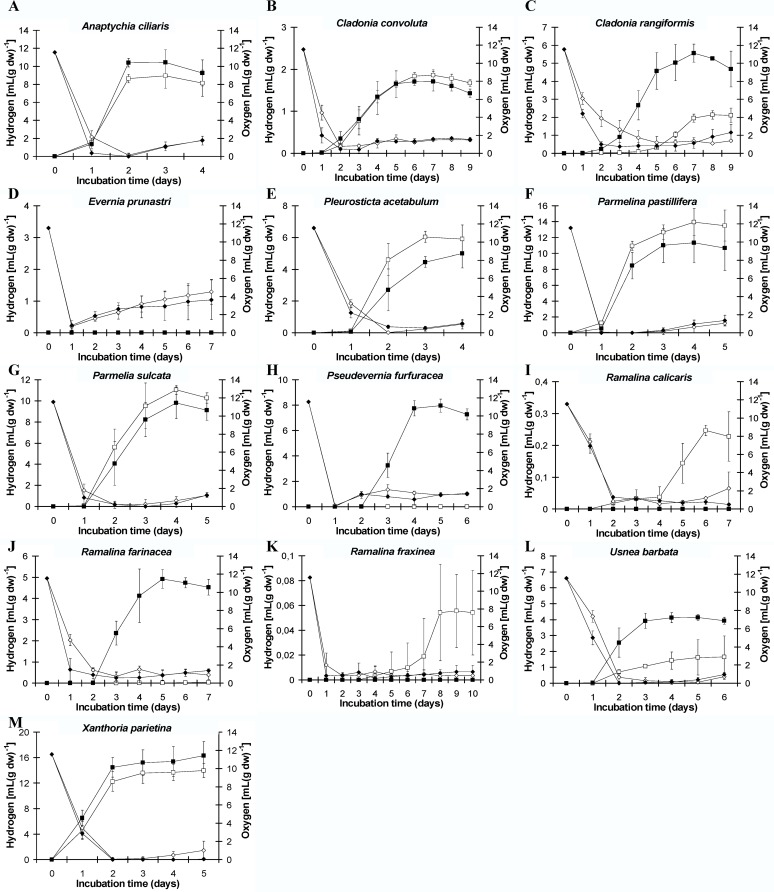
Kinetics of hydrogen production and oxygen consumption under light (white shapes) and dark incubation (black shapes) in (A) *Anaptychia ciliaris*, (B) *Cladonia convolute*, (C) *Cladonia rangiformis*, (D) *Evernia prunastri*, (E) *Pleurosticta acetabulum*, (F) *Parmelina pastilifera*, (G) *Parmelia sulcata*, (H) *Pseudevernia furfuraceae*, (I) *Ramalina calicaris*, (J) *Ramalina farinacea*, (K) *Ramalina fraxinea*, (L) *Usnea barbata*, (M) *Xanthoria parietina*.

More information about the kinetics of hydrogen production and oxygen consumption of the tested species appear in Fig. 9.

The majority of the lichen species tested produced hydrogen under dark and light conditions. A variety of incubation times were required for the induction of hydrogenase, which usually correlated with the consumption of oxygen. The different ratio of mycobiont to photobiont biomass in each species, their different evolutionary origin, their occurrence in various natural habitats, their different thallus morphology or their different shape, texture and pigmentation could be some of the parameters that affected the final hydrogen production under the tested experimental conditions.

Among them *Ramalina calicaris* (Fig. 9I) and *Ramalina fraxinea* (Fig. 9K) produced hydrogen only in light conditions (very close to zero value), while *Ramalina farinacea* (Fig. 9J) produced hydrogen mainly in dark and very low quantities in light conditions as did the species *Ramalina fraxinea* (Fig. 9K) and *Ramalina calicaris* (Fig. 9I). *Evernia prunastri* (Fig. 9D) cannot produce hydrogen in the tested experimental conditions neither in the light nor in the dark. *Xanthoria parietina* (Fig. 9M) seems to be the best species concerning hydrogen production under dark conditions (approximately 16.3 mL H_2_ per g of dry weight), while *Xanthoria parietina* (Fig. 9M) and *Parmelina pastillifera* (Fig. 9F) were those most efficient under light incubation (approximately 14 mL H_2_ per grams of dry weight.

*Anaptychia ciliaris* (Fig. 9A), *Parmelia sulcata* (Fig. 9G), *Parmelina pastillifera* (Fig. 9F) and *Xanthoria parietina* (Fig. 9M) produced higher hydrogen production compared to *Pleurosticta acetabulum* (Fig. 9E) under light conditions, while *Cladonia convoluta* (Fig. 9B), *Cladonia rangiformis* (Fig. 9C), *Usnea barbata* (Fig. 9L) followed with lower values. Finally, the hydrogen production measurements of *Ramalina calicaris* (Fig. 9I), *Ramalina fraxinea* (Fig. 9K) *and Pseudevernia furfuracea* (Fig. 9H) were extremely low.

In dark conditions, *Pseudevernia furfuracea* (Fig. 9H), *Anaptychia ciliaris* (Fig. 9A), *Parmelia sulcata* (Fig. 9G), *Parmelina pastillifera* (Fig. 9F) *and Xanthoria parietina* (Fig. 9M) produced higher hydrogen compared to *Pleurosticta acetabulum* (Fig. 9E). *Cladonia rangiformis* (Fig. 9C), *Ramalina farinacea* (Fig. 9J), *Usnea barbata* (Fig. 9L) produced hydrogen similar to the lichen *Pleurosticta acetabulum* (Fig. 9E), while *Cladonia convoluta* (Fig. 9B) produced extremely lower quantities.

## Discussion

**Fig 10:**
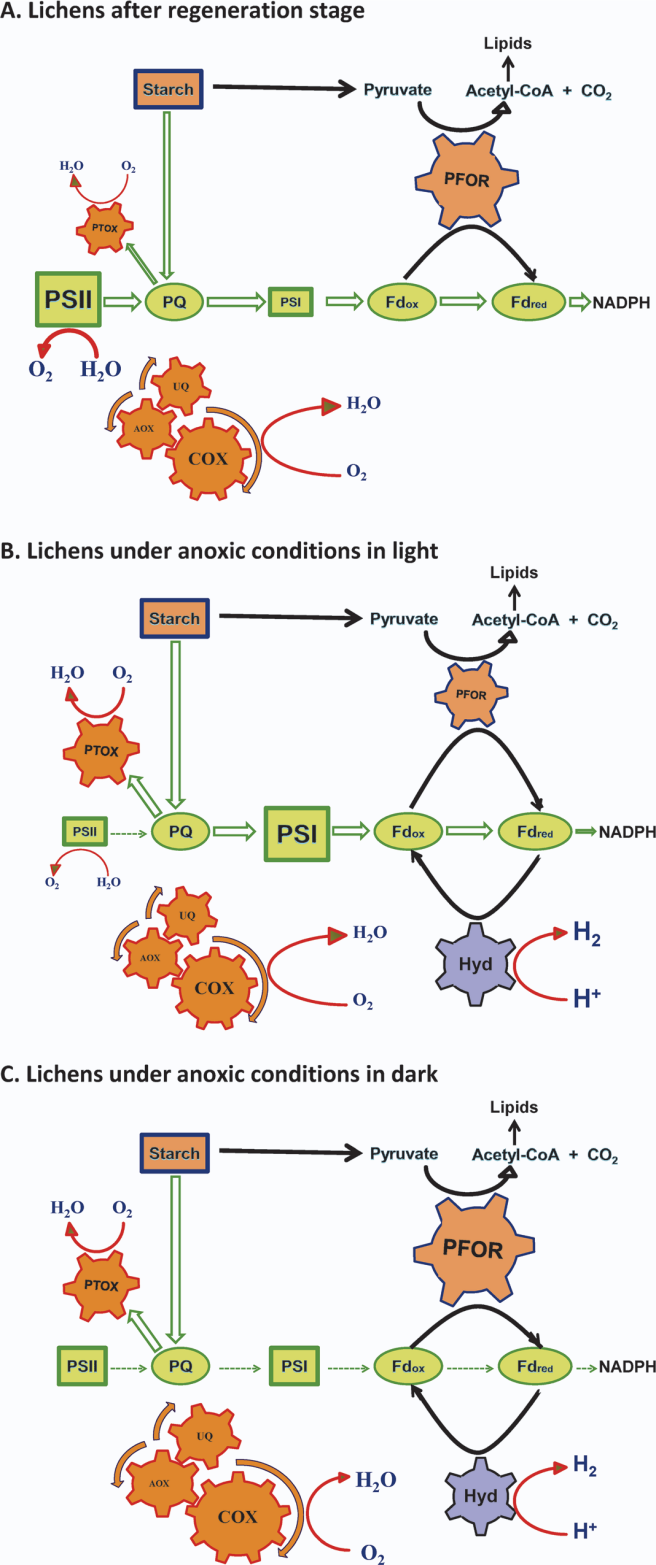
Proposed models for hydrogen production of the lichen *Pleurosticta acetabulum*. (A) Lichens exactly after the regeneration stage. (B) Lichens under anoxic conditions in light. (C) Lichens under anoxic conditions in dark.

This is the first report that highlights the symbiotic syntheses of lichens (mycobiont + phycobiont) as bio-factories that are able to achieve high yields of hydrogen production. Lichens are naturally occurring organisms that under specified conditions have the capability to produce hydrogen in high yields (Figs. 8A and 9). The symbiotic relationship between a mycobiont (oxygen consumer) and a photobiont (with three possible pathways for H_2_ production under oxygen depleted conditions) is the basic advantage of lichens in hydrogen production. The consumption of oxygen (Figs. 8B and 9) mainly by the mycobiont in a closed system creates the appropriate hospitable environment for the activation of the O_2_-sensitive chloroplastic hydrogenase from the photobiont (Fig. 7F). Hydrogenase receives electrons from ferredoxin. The origin of the above electrons was the photosynthetic splitting of water (PSII-dependent pathway) [6–9], the reduction of glucose (PSII-independent pathway) [10,11] (Fig. 10B) and the dark fermentative pathway through pyruvate ferredoxin oxidoreductase [12] (Figs. 7G, 10B-C).

The paradox is that several attempts (continuous nitrogen, argon or helium flow [14], sulfur [38], nitrogen [16] or potassium deprivation [17], addition of *meta* substituted dichlorophenols to the culture medium [18,19] or genetically modified organisms [39]) have been investigated in the field of hydrogen production for overcoming the inhibition of hydrogenase in the presence of oxygen. However, the solution has already existed in nature for millions of years and can be found in the biochemical mechanisms of lichens. Also, it is worth mentioning that the detected hydrogen values, when taking into consideration the extremely low algae density in lichens, are extremely higher compared to cultures exclusively containing algae.

The majority of the parameters that affected hydrogen production in algae had a similar impact on the lichens tested. Glucose is an important exogenously supplied carbon source that improves hydrogen production. Higher productivities were accomplished when glucose was added in the stage of incubation instead of the regeneration stage (Fig. 2A, C and E). These results are in absolute agreement with our previous publications based on the hydrogen production of the green alga *Scenedesmus obliquus* [17–19].

The volume of the medium in the hermitically closed bottles also seems to plays a crucial role in hydrogen release, due to the partial pressures in the liquid-air interface. The lower the medium volume the higher the hydrogen productivity detected (Fig. 3). The same phenomenon had previously been observed in hermitically closed cultures of *Scenedesmus obliquus* and has been analytically explained [17].

Temperature is another abiotic parameter that maximizes hydrogen production. However, there is an ideal temperature for the activation of hydrogenase [31,32] which in our case was 30°C (Fig. 5A). The above observations are quite similar to the results reported for the fermentative hydrogen production of mixed cultures, using exogenously supplied glucose as a substrate [32].

The composition of the medium does not significantly affect the quantity of the hydrogen produced, maybe because of the lichens’ nature, and so the choice of using deionized water enriched with 5 g L^-1^ of glucose appeared more reasonable (Fig. 4).

The light intensity (low, medium or high) does not affect the lichen’s hydrogen generation, but the presence or absence of light has a significant effect on the released hydrogen since different pathways are activated (Figs. 6 and 10). These results are totally different compared to our recently published data, based on the influence of light intensity on the hydrogen productivity of the green alga *Scenedesmus obliquus* [17]. In these treatments the light intensity affected the measured hydrogen values. However, the algal density was extremely higher compared to the lichens’ algal density and the produced hydrogen was mainly due to the light induced hydrogen pathways (PSII-dependent and PSII-independent pathways), in opposition to the majority of the lichens, where the dark fermentative pathway was fully activated (Figs. 7G, 10B-C).

All the specific conditions mentioned, were identified after the experimental testing of the lichen species *Pleurosticta acetabulum* and used for scanning a wide range of other lichen species. These trials support that the ability of lichens to produce hydrogen is a general aspect and is not limited only to the lichen species *Pleurosticta acetabulum* (Figs. 8 and 9).

The proposed mechanism for hydrogen production in the above mentioned conditions is explained in a simplified model for the species *Pleurosticta acetabulum* (Fig. 10). Fig. 10A refers to the time point exactly after the regeneration of the lichens, Fig. 10B to the oxygen depleted conditions under light incubation and Fig. 10C to the oxygen depleted conditions under dark incubation.

In Fig. 10A, where the processes exactly after regeneration are presented, the PQ pool is fed with electrons mainly due to the light induced water splitting in PSII and incidentally due to the reduction of glucose (endogenously or exogenously supplied). The above electrons are transferred to ferredoxin through PSI (photosynthetic electron transport chain) or through PFOR (dark fermentation) for NADPH production, lipid and growth increase. In parallel to the photosynthetic electron transport chains, there are mechanisms for consuming oxygen and producing energy, such as the cytochromic (Fig. 7A) and alternative mitochondrial (Fig. 7B) pathways and chlororespiration (Fig. 7C). Under these conditions, the oxygen concentration remains high and as a result the hydrogenase activity was totally inhibited (Fig. 7F).

In Fig. 10B, where the processes during light incubation are presented, as the lichens are placed in the hermitically closed bottles, anoxic conditions are established, because of the over activation of oxygen consumption through the respirational electron transport chains (in mitochondria and chloroplasts—Fig. 7A-C). The oxygen depleted conditions in combination with the inactivation of PSII (Fig. 7E) create the optimal conditions for hydrogen generation. Specifically, PSII was deactivated and the remaining electrons were transferred from PSI to ferredoxin and then to hydrogenase (PSII-dependent pathway). The hydrogen production was further induced by the reduction of organic substrates through the PSII–independent pathway. These electrons are led to the plastoquinone pool and through PSI (hyperactivation of PSI—Fig. 7D) and ferredoxin, are transferred to hydrogenase for hydrogen production. In parallel, electrons are alternatively allocated to pyruvate and through the PFOR protein (Fig. 7G) result in ferredoxin and hydrogenase. It is obvious that under light incubation the PSII-dependent and the PSII-independent pathways are more active than the dark fermentative one.

On the contrary, in Fig. 10C that corresponds to dark incubation conditions, the dark fermentative pathway is the dominant route for feeding ferredoxin with electrons (Fig. 7D-G), since the other two pathways are absolutely deactivated due to the absence of light. In dark conditions, oxygen consumers are more active (Figs. 7A-C and 9), as was expected, and the hydrogen production was observed at earlier incubation times (Fig. 9).

In conclusion, all the experiments clearly demonstrated that lichens could be nature's solution for overcoming the problem of the O_2_ sensitive hydrogenase. Lichens can establish anoxic conditions in a closed system mainly through O_2_ consumption by the mycobiont and in parallel produce high yields of hydrogen by the photobiont using three different pathways (PSII-dependent, PSII-independent and dark fermentation). They have the ability to activate the appropriate bioenergetic pathways under anaerobic conditions in order to produce hydrogen. Depending on the specific incubation conditions they can efficiently use either light induced hydrogen production or dark fermentation. The above mentioned benefits of lichens in combination with their ability to survive in extreme environments [40] constitute them as invaluable future hydrogen production factories, applicable even in space. These properties will render lichens as very important organisms in the field of biohydrogen-energy production. Further investigation is required in order to pave the way for future sustainable biotechnological applications, even in an industrial scale use.

## Materials and Methods

### Lichen species

*Pleurosticta acetabulum* is a green algae lichen with a dark olive-green to brown-grey (oily green when wet) thallus, closely appressed and spreading, wrinkled towards the center, with upturned lobe ends which appear dotted with dark spots. Lower surface is pale brown to black. Soredia or isidia, which act as vegetative propagules are absent, while large apothecia (to 15 mm diam.) with red-brown discs and margins in-rolled are common. Spot tests: medulla K+ red, C-, Pd +orange.

*Pleurosticta acetabulum* and *Xanthoria parietina* samples have been collected from *Acer sempervirens* and *Quercus coccifera* substrates of Mountain Idi (HTRS07/TM07 X = 578576.83 m, Y = 1900901.19 m). *Cladonia convoluta* and *Cladonia rangiformis* have been collected from a Mediterranean shrubland area of Northern Greece, close to the city of Kilkis (HTRS07/TM07 X = 402270.42 m, Y = 2540414.83 m). Finally, the remaining species (*Ramalina fraxinea*, *Ramalina farinacea*, *Ramalina calicaris*, *Pseudevernia furfuracea*, *Evernia prunastri*, *Parmelia sulcata*, *Parmelina pastillifera*, *Anaptychia ciliaris*, *Usnea barbata)* have been collected from deciduous substrates (mainly *Quercus* trees) of Mountain Kerkini (Belles) (HTRS07/TM07 X = 436101.75 m, Y = 2576812.6 m). All GPS coordinates follow the Hellenic Terrestrial Reference System 2007 (HTRS07/TM07).

The permission to collect the lichen samples was granted by the Hellenic Ministry of Environment Energy and Climate Change, Special Secretariat for Forests (No 108436/956) in accordance with the national law (N3937/2011) on biodiversity conservation. All the collections derived from public lands and from areas where no other specific permissions were required (Article 17), since the wildlife of these areas is not protected by other local/regional regulations or national law. Moreover, the field studies did not involve endemic, endangered or protected species.

### Regeneration protocol for lichen samples

In total 2 g (dry weight) of the lichen tissue was washed with deionized water and submerged for 10 minutes. The lichen tissue was wrapped in a wet paper towel, with plenty of moisture for the duration of the regeneration. The packaged lichen sample remained at room temperature for 1 hour and then was transferred to a cold room (4–6°C) without lighting overnight. The quality of regeneration was tested with fluorescence induction measurements and the JIP-test (see following). Only if the tissue had F_v_/F_m_ (maximal photosynthetic efficiencies) values higher than 0.7 it was used for the experimental treatments.

### Experimental procedure for lichen samples

After regeneration the lichens were removed from the cold room (4–6°C) and placed in a sterile environment. The lichens samples were carefully transferred into 125 mL hermitically sealed bottles (diameter 5 cm, height 9.5 cm) with an initial volume of 10 mL sterilized medium solution (deionised water with 5 g L^-1^ glucose). Then, the bottles were placed sideways in a temperature-controlled chamber (30°C) at a light intensity of approximately 20 μE. The above conditions were the usual ones and any changes are explained in detail in the appropriate subsection of the results.

### GC-TCD measurements of H_2_ and O_2_

Hydrogen and oxygen measurements were made utilizing gas chromatography, using a thermal conductivity detector (GC-TCD) (Shimadzu GC 2010 Plus, Kyoto, Japan). To separate H_2_ and O_2_, argon was used as the carrier gas under pressure of five bars and at oven temperature of 120°C. The column used was a capillary Vici Metronics MC (Poulsbo, USA) with length 30 m (diameter: 0.53 mm) and film thickness 20 μm. The temperature of TCD was set at 200°C for the detector and 180°C for the injector. The quantification of hydrogen and oxygen was done by injecting known quantities in the GC-TCD [18].

### Fluorescence induction measurements

The Handy Plant Efficiency Analyser, PEA (Hansatech Instruments, Kings’s Lynn, Norfolk, UK) was used for the fluorescence induction measurements ensuring the success of the regeneration process and the good quality of lichen tissue for the designed experiments. The maximum yield of photochemistry (F_v_/F_m_) was the main index used for the physiological state of lichen tissues and measured according to the JIP method of Strasser and Strasser [41]. This method is based on the measurement of a fast fluorescence transient with a 10 μs resolution in a time span of 40 μs to 1 s. Fluorescence was measured at 12-bit resolution and excited by three light-emitting diodes providing a saturated light intensity of 3000 μE of red (650 nm) light. The Handy PEA data sampling operates at a maximum frequency of 100 kHz only for the first 300 μs and then the frequency decreases.

### Protein extraction and quantification

Total proteins were extracted according to the method of Siminis et al. [42]. Protein determination was performed according to Lowry et al. [43].

### Western blotting

Protein extracts (50 μg total protein) were electrophoretically resolved using SDS-PAGE, transferred to membranes and hybridised against several antibodies according to Agrisera protocols: 10% SDS PAGE for PsaA (Agrisera), HydA (Agrisera) and PFOR. 13% SDS PAGE for D_1_ protein. 12% SDS PAGE for COX, AOX and PTOX. The quantification of western band proteins took place using the imageJ program and were expressed as % relative intensity of the control.

### Data analysis

Each experiment was repeated at least three times and each treatment included five independent samples. Standard deviations of the average values are presented on the diagrams.
